# A Review of Gelatin Source Authentication Methods

**DOI:** 10.21315/tlsr2018.29.2.15

**Published:** 2018-07-06

**Authors:** Ademola Monsur Hameed, Tawakalit Asiyanbi-H, Munirat Idris, Nurrulhidayah Fadzillah, Mohamed Elwathig S. Mirghani

**Affiliations:** 1 International Institute for Halal Research and Training, International Islamic University Malaysia, 53100 Gombak, Kuala Lumpur; 2 Plant Science Department, North Dakota State University, Fargo, North Dakota, United States

**Keywords:** Gelatin, Halal, Authentication, Proteomics, Peptides-Marker, Gelatin, Halal, Pengesahan, Proteomik, Penanda Peptida

## Abstract

Gelatin is a very popular pharmaceutical and food ingredient and the most studied ingredient in Halal researches. Interest in source gelatin authentication is based on religious and cultural beliefs, food fraud prevention and health issues. Seven gelatin authentication methods that have been developed include: nucleic acid based, immunochemical, electrophoretic analysis, spectroscopic, mass-spectrometric, chromatographic-chemometric and chemisorption methods. These methods are time consuming, and require capital intensive equipment with huge running cost. Reliability of gelatin authentication methods is challenged mostly by transformation of gelatin during processing and close similarities among gelatin structures. This review concisely presents findings and challenges in this research area and suggests needs for more researches on development of rapid authentication method and process-transformed gelatins.

## Abstract (ms)

Gelatin adalah bahan farmaseutikal dan makanan yang sangat terkenal serta menjadi ramuan yang paling banyak dikaji dalam bidang penyelidikan Halal. Asas galakan untuk diadakan pengesahan (autentikasi) sumber gelatin adalah disebabkan kepercayaan agama dan budaya, pencegahan penipuan makanan dan isu kesihatan. Tujuh kaedah pengesahan (autentikasi) gelatin yang telah dilaksanakan termasuk kaedah: berasaskan asid nuklik, imunokimia, analisis elektroforetik, spektroskopi, spektrometrik massa, kaedah kromatograf-kimometrik dan serapan kimia. Kaedah-kaedah ini memakan masa dan memerlukan peralatan bermodal intensif dengan kos yang besar. Keberkesanan kaedah pengesahan (autentikasi) gelatin adalah suatu cabaran besar disebabkan oleh transformasi gelatin semasa pemprosesan dan persamaan rapat diantara struktur gelatin. Kajian ini mengulas secara ringkas penemuan-penemuan dan cabaran-cabaran dalam bidang penyelidikan ini dan menyediakan cadangan untuk lebih banyak penyelidikan mengenai perkembangan kaedah pengesahan (autentikasi) yang pantas dan gelatin yang telah berubah akibat pemprosesan.

## INTRODUCTION

As at 2013, the global Halal market size was about 580 to 660 billion US Dollars and has great potential to increase in the future year (Jahangir *et al.* 2016). Apart from increasing Muslim population and Halal awareness, Halal market is driven by development of Halal ingredients authentication methods that strengthening consumers’ confident (Malik *et al.* 2016). Gelatin is among the most studied Halal ingredient because of its vast usage in pharmaceutical and food products. Gelatin is a hydrocolloid with unique properties and can function as gelling, thickener, foaming agent, plasticizer, texture and binding agent (Sahilah *et al.* 2012).

The sources of most commercial gelatin are from mammalian (bovine and mostly porcine) bone and hide (Shabani *et al.* 2015). Consumers’ concern about gelatin source is due to cultural and religious beliefs. For examples, Muslims and Jews reject porcine based food derivatives, Chinese traditional medicine use only gelatin from donkey skin for treatment of some disease (Nemati *et al.* 2004) and vegetarians avoid animal based products. Increase in Halal awareness among Muslims has called for great need of food-source authentications. Gelatin and gelatin based products are currently classified as doubtful because haram (porcine) gelatin is the most abundant. Traceability of gelatin source has been a great task in Halal field. Other reasons for gelatin source authentication include: (i) protection of consumer from food frauds due to false labeling and avoid unfair market competition (Kesmen *et al.* 2009) and (ii) health issues. The use of bovine carrying prion proteins has been linked to bovine spongiform encephalopathy (mad cow disease) causing immune response that led to fatal neurodegenerative disease (spongy degeneration of the brain and spinal cord) in cattle (Widyaninggar *et al.* 2012). Also, bovine and porcine gelatins is risky to gelatin-allergic patients (Raraswati *et al.* 2013).

Gelatin contains protein, peptides or nucleic acids (Malik *et al.* 2016). Proteins, peptide derivatives and nucleic acids are favorable means for tissue identification in samples even after it has undergone heating, for instance, in processed food (Buckley *et al.* 2009). However, large similarities between structures of gelatin from different sources make their differentiation difficult (Nemati *et al.* 2004). Many gelatin source authentication methods have been developed including: spectroscopic method (Hashim *et al.* 2010; Hermanto & Fatimah 2013), immunochemical method (Tukiran *et al.* 2015, 2016a), nucleic acid based method (Cai *et al.* 2012; Demirhan *et al.* 2012; Malik *et al.* 2016; Mutalib *et al.* 2015; Shabani *et al.* 2015; Sudjadi *et al.* 2015), mass spectrometric method HPLC/MS (Yilmaz *et al.* 2013; G.-F. Zhang *et al.* 2008; G. Zhang *et al.* 2009), chromatographic-chemometric method (Azilawati *et al.* 2015), electrophoretic analysis (Azira *et al.* 2014) and chemisorption (Hidaka & Liu 2003).

We have reviewed gelatin source authentication methods and identified their achievements and challenges. Current gelatin source authentication methods are time consuming and expensive, thus required development of cheap and quick analytical methods. Likewise, gelatin transformation during processing can potentially hinder the efficiency of the currently developed gelatin authentication methods. This work will serve as a quick reference guide for future researchers.

## AUTHENTICATION OF GELATIN SOURCES

As earlier stated, several methods have been developed to investigate sources of gelatin in commercial gelatin or gelatin-containing products. Authentication of gelatin sources in several products have been investigated (Table 1). Researchers have adopted several approaches to develop specie-specific gelatin authentication methods. The approaches include use of the amino acid profile, the presence of specific peptide and DNA markers and the spectral fingerprint of gelatins or its peptides.

### Nucleic Acid Based Method

The DNA techniques can be used for qualitative and quantitative analysis of processed food because of their stability. The technique can be used virtually in all samples because of the ubiquity of DNA. This technique is highly specific, very sensitive (up to 0.1%) and capable of detecting a small amount of certain DNA in the sample (Wolf & Lüthy 2001). Other advantages of DNA-based technique are less contamination risk and larger dynamic range of detection (Kesmen *et al.* 2009). More so, the Polymerase chain reaction (PCR) techniques can be monitored by fluorescence thus eliminate detection steps (Kesmen *et al.* 2009).

Table 2 depicts the characteristics of bovine and porcine primers previously used in Halal authentication of gelatin source. Although universal primer can be used, a specific primer which allows shorter amplicons is better for food samples. This is because universal primer requires long PCR products with adequate DNA sequence variation for restriction analysis or single strand conformation polymorphism and thus not suitable for food samples that usually contain degraded DNA with few hundred base pairs (Wolf & Lüthy 2001).

In the work of Cai *et al.* (2012) two species-specific quantitative-PCR (qPCR) assays was developed for the detection and quantitation of porcine and bovine DNA in gelatin blends and gelatin capsules. The developed assay was highly specific as it lacks cross-reactivity during amplification of each samples DNA. Also, the assay was highly sensitive to detect contamination level as low as 1.0% in gelatin blends (bovine + porcine). Analysis of gelatin capsules revealed possible discriminative detection and quantification of porcine and bovine species identities, although, less DNA was detected.

In another study, Demirhan *et al.* (2012) reportedly used real-time PCR techniques to authenticate the source of gelatin in gum drops, marshmallows and Turkish delight. Two of 14 products from Germany were positive for porcine DNA whereas only 1 of 29 products from Turkey was positive for porcine DNA. Also, Sahilah *et al.* (2012) used PCR and southern hybridisation on biochip analysis to investigate soft and hard gel capsules for Halal compliance. The result showed that 42 (without Halal logo) out of 113 capsules tested positive for porcine DNA. This evinced the suitability of PCR techniques and southern hybridisation on the biochip for monitoring haram compounds in the soft and hard capsule.

Furthermore, Mutalib *et al.* (2015) also used PCR-southern hybridisation on chip and convention PCR for Halal authentication of gelatin in 20 capsule brands. PCR-southern hybridisation on chip detected DNA in 6 capsules that were undetected by conventional PCR analysis. Compared to conventional PCR, PCR-southern hybridisation on the chip was more suitable and dependable for authenticating porcine DNA in gelatin capsules. Shabani *et al.* (2015) reportedly used species-specific PCR technique to validate gelatin sourced from 8 gelatin containing food products (two marshmallow, two jellies, two deserts and two cakes) and observed them to be from bovine but not porcine. Sudjadi *et al.* (2015) also used real-time PCR technique to validate the lack of porcine DNA in the capsule shell.

### Immunochemical Method (Enzyme-linked immunosorbent assay, ELISA)

The principle of enzyme-linked immunosorbent assay (ELISA) is based on the binding effect of the antibody with specific macromolecular analytes – antigens – such as protein/peptide (Ale *et al.* 2011). Alternatively, the antibody might be the analytes rather than an antigen. In addition, a measurable signal is produced through the introduction of chemically linked antibodies or antigen with a detectable label in order to allow for measurement (Ekins 1991). The reporter labels that have been utilised in ELISA are basically enzymes such as horseradish peroxidase, alkaline phosphatase and glucose oxidase (Lequin 2005).

ELISA method is advantageous because of structural specificity, detectability, sensitivity and high sample throughput capacity (Ekins 1991; Martín *et al.* 2009). It equally offers simplicity in term of sample preparation as high purity is not necessary. Thus, immunochemical differentiation is a valuable alternative method for authentication of gelatin source (Tukiran *et al.* 2016b).

Recent research efforts on the development of ELISA technique for gelatin source authentication have been reported. Briefly, the experimental procedure involves (i) identification of suitable specie-specific sequence (from gelatin peptides) (ii) production of synthetic peptides (iii) crosslinking of peptides with keyhole limpet hemocyanin (iv) immunisation of rabbit (iv) recovery of produced of polyclonal antibodies (v) development of an indirect and/or competitive indirect ELISA. However, there might be slight variation in the literature.

Venien and Levieux (2005) developed indirect and competitive indirect ELISA using polyclonal anti-peptide antibodies for differentiation of bovine from porcine gelatins. It was reported that competitive indirect ELISA was more appropriate to detect bovine gelatin in porcine gelatin to a dilution of 2–4 parts per 1000. Tukiran *et al.* (2015) also developed indirect ELISA using anti-peptide polyclonal antibodies for porcine gelatin detection in dibble bird’s nests. The result showed that the developed ELISA was able to detect at least 0.5 ng/μg porcine gelatin in spiked samples. In another study, Tukiran *et al.* (2016b) developed indirect ELISA using anti-peptide (collagen α2) polyclonal antibodies for authentication of gelatin source in confectionery products. The developed ELISA showed low cross-reactivity to fish and chicken gelatin and the detection limit was 0.05 μg/mL. Furthermore, Tukiran *et al.* (2016a) also developed competitive indirect ELISA based on the anti-peptide polyclonal antibody for determination of porcine gelatin in edible bird’s nest. Among the three porcine species-specific peptides, the sequence that used collagen α1 was found to be sufficient for authentication of edible bird’s nest with a detection limit of 0052 μg/mL.

The challenge of immunochemical method is lack of antibodies stability in alkaline or acidic process which is commonly used for gelatin production (Yilmaz *et al.* 2013). Also, ELISA method lack enough species-specific detection of mixture containing porcine gelatin and low concentration of bovine gelatin (Hashim *et al.* 2010).

### Electrophoretic Analysis (Sodium dodecyl sulfate-polyacrylamide gel electrophoresis, SDS-PAGE)

Sodium dodecyl sulfate-polyacrylamide gel electrophoresis (SDS-PAGE) technique is used for separation of proteins with different molecular weight (Hamdan & Righetti 2005). Molecular weight is among the factors that affect the functional quality of gelatin. The variation in molecular weight distribution of protein/peptide fractions among different sources of gelatin can be used as a basis for authentication of gelatin source (G. Zhang *et al.* 2009). Commercial gelatins contain different protein fractions including *α* (100 kDa), *β* (200 kDa) and *γ*-chains (300 kDa) (Z. Zhang *et al.* 2006). Polypeptide molecular weight distribution is also affected by processing methods and the level of hydrolysis that take place (Karim & Bhat 2009).

Hermanto and Fatimah (2013) studied the differentiation of bovine and porcine gelatin and its peptides using SDS-PAGE techniques. Although the molecular weight distribution of gelatin and its fragments was successful, no real differentiation was achieved except for gelatin from porcine after 2 h of pepsin hydrolysis in which two peptide fragments were observed with molecular sizes below 36.2 and 28.6 kDa.

Azira *et al.* (2014) used SDS-PAGE combined with densitometry (principal component analysis) for differentiation of gelatin sources (bovine and porcine). Polypeptides with a molecular weight ranging from 50 to 220 kDa exhibited differing intensities when separated using SDS-PAGE. Principal component analysis of molecular weight distribution of electrophoretic polypeptide patterns was able to detect 5% porcine gelatin in bovine gelatin mix.

Another study by Malik *et al.* (2016), SDS-PAGE coupled with densitometry analysis was also used to differentiate porcine and bovine gelatin in capsule shells. Twelve major bands of peptide profile were observed in porcine gelatin as against bovine gelatin that only showed four major bands.

### Spectroscopic Methods (UV-Vis and Fourier-transform infrared spectroscopy, FTIR)

Spectroscopic measurements are very sensitive, nondestructive and require a small amount of sample with little or no sample preparation. UV-Visible spectroscopy is used to measure the absorption of high-energy light (wavelengths of 200–800 nm) that causes excitation by molecules containing conjugated pi-electron system in a covalent bond. Biological macromolecules contain delocalised electron in aromatic systems that often absorb light in the near-UV (150–400 nm) or the visible (400–800 nm) region (Aitken & Learmonth 2002; Schmid *et al.* 2001). Molecular origin of protein absorbance includes the peptide groups in the far-UV (180–230 nm), the aromatic side chains of Tyr, Trp and Phe absorb light in the far-UV (180–230 nm) region and the near-UV (240–300 nm) region and the disulfide in near-UV (260 nm) (Aitken & Learmonth 2002; Schmid 2001).

Infrared spectroscopy is one of the most powerful spectroscopic methods to generate infrared spectrum which contained details of the functional group and chemical composition of food compounds (Barth 2007; Hermanto & Fatimah 2013). Infra-Red (IR) spectroscopy measures the amount of light absorbed as a result of molecular vibrations over a range of frequencies of the incident light. FTIR spectroscopy has been used to study the changes in secondary structure of collagen and gelatin, collagen crosslinking, thermal self-assembly and gelatin melting (Hermanto & Fatimah 2013). Spectra changes in amide A, amide I, amide II and the amide III regions have been observed in collagen secondary structures. FTIR spectroscopy is a valuable technique for determination of adulteration in food products including gelatin (Barth 2007). Use of FTIR for differential analysis of intramolecular structures of gelatin from different sources is a viable approach for Halal authentication practice (Hermanto & Fatimah 2013).

Hashim *et al.* (2010) has established the potential use of FTIR in combination with attenuated total reflectance and principal component analysis for differentiation of bovine and porcine gelatins. FTIR spectra in the range of 3290–3280 cm^−1^ and 1660–1200 cm^−1^ were identified and used as calibration model. A clear difference was obtained from the result for PCA represented by the Cooman’s plot. Information from FTIR spectra provide rapid determination of the gelatin source and serves as a base for second derivative study.

Hermanto and Fatimah (2013) studied the difference between pepsin hydrolysates of bovine and porcine gelatin using UV-Vis and FTIR spectroscopy. In UV-Vis spectroscopy, both gelatin sources had a different absorbance at 229 nm and 240 nm showing the proportion of C=O amide and differences in the two-dimensional conformation of the peptide. The three differences in FTIR spectra of the hydrolyzates after 1 h of hydrolysis that were observed are the region 2800–3000 cm-1 (aliphatic C-H stretching), the region 1543 cm^−1^ (C-N-H bending) and in the frequency of 1450–1300 cm^−1^ (C-H bending). The third difference was associated with a difference in the amino acid profile of the two samples.

Although, spectroscopic methods were reportedly suitable for gelatin source authentication, data should be repeatable in order to obtain reliable information (Yilmaz *et al.*, 2013). Likewise, differentiation of processed-transformed gelatin from different sources is still a challenge.

### Mass Spectrometric Method

Mass spectrometry (MS) has been found to be very useful for qualitative and quantitative analysis of protein and peptides. Although, it was initially developed to study small molecules, several improvements have recently led to the use of MS in proteomics. These developments have led to different types of MS such as time-of-flight (ToF), quadrupole, (Paul) ion trap, FTICR, and orbitrap, as well as, different techniques (matrix-assisted laser desorption/ionization (MALDI) and electrospray ionisation (ESI) that allowed the ionisation of biomolecules (peptides, proteins, and nucleotides) for easy detection by mass spectrometry. Proteomic studies involve a mixture of numerous proteins/peptides that are required to be separated using chromatography techniques such as high-performance column chromatography (HPLC) and ultra-performance column chromatography (UPLC). Hybridisation of different mass spectrometers (MS) combined with HPLC/UPLC, MALDI/ESI has made mass spectrometric methods for protein quantification become more powerful (Nikolov *et al.* 2012). Several studies have been conducted to developed MS-based methods for differential analysis of gelatin source.

Ocaña *et al.* (2004) investigated the use of MALDI-TOF MS and LC-ESIMS-MS for detection of HCl gelatin-derived peptides in animal feed. The derived hydrolytic peptides at m/z 828, 915, 957 and 1044 were analysed by MALDITOF MS and LC-ESI-MS-MS. Using MALDI, the sensitivity of the method was reportedly improved to 100 ng mL^−1^ of peptide solution. It was concluded that some species-specific ions could be used as a marker for the presence of bovine gelatin in animal feed.

G.-F. Zhang *et al.* (2008) and G. Zhang *et al.* (2009) utilised high-performance liquid chromatography/mass spectroscopy (HPLC/MS) and HPLCMS/MS, respectively, to analyse trypsin digested bovine and porcine gelatins. Specie-specific (maker) peptides were identified and their sequences could be used for differential analysis of bovine and porcine gelatins. The maker peptides contained hydroxylated prolines that should be considered to increase the confidence of peptide identification. This is because of the possibility of mass shift due to proline hydroxylation can be misleading.

Buckley *et al.* (2009) conducted species identification of bone collagen peptides from 32 different mammal species using MALDI-TOF MS. The peptide obtained using solid-phase extraction showed 92 peptide markers that could be used for species identification in processed food and animal feed.

Yilmaz *et al.* (2013) presented an ultra-performance liquid chromatography and electrospray ionisation quadrupole time-of-flight mass spectrometry (nanoUPLC-ESI-Q-TOF-MS) based data independent acquisition technique to detect maker peptides for differential analysis of gelatin sources (porcine and bovine) in dairy products. Specie-specific peptides maker were successfully detected in gelatin added to dairy products analysed. The result showed that nanoUPLC-ESI-Q-TOF-MS method is an effective and a rapid way to determine the gelatin origin.

Grundy *et al.* (2016) presented a method to determine the species origin of gelatins by peptide-MS methods (MALDI-TOF and LC-ESI-MS-MS). The peptides were obtained by pepsin digestion of nine commercial gelatins. It was found that the commercial gelatins contained undeclared species and that the method was able to determine not only the origin of gelatin but was also able to differentiate between bovine and porcine as well as other wide range of species (horse, goat, sheep, fish and various poultry species). Hence, it was concluded that the method could support food industry in determining the species authenticity of gelatin in foods.

However, the drawbacks of mass spectrometric method include (i) difficulties in detection of peptides marker in digested gelatin due to high level of similarities between gelatin sequence in mammals, (ii) proline hydroxylation pose challenge that make peptide identification more difficult and (iii) the threshold for specific peptide identification varies from species to species (Yilmaz *et al.* 2013).

### Chromatographic-Chemometric Method

High-performance liquid chromatography (HPLC) has become a very useful analytical technique for separation of biomolecules based on differences in their structure and/or composition (Kupiec 2004). Reverse phase HPLC (RP-HPLC) is the most commonly used because it is easier to use, suitable for wide range of molecules and readily controlled. For high sensitivity, detection with fluorometer is more suitable for amino acid analysis with RP-HPLC. Therefore, most HPLC methods required derivatisation of amino acids to make them fluorescence (Sander & Wise 1987).

Chemometric which is also known as Principal component analysis (PCA) is the unsupervised pattern recognition technique for classification and differentiation of objects (Ziegel 2004). PCA facilitate data restructuring by reducing the dimension of data to separate samples from different groups. PCA is based on the mathematical and statistical operations to distinguish the variables of analogous profiles. Prior to running PCA task, data are pretreated using Normalisation method to improve data sets that contained high variation (Azilawati *et al.* 2015).

The combination of the chromatographic and chemometric method has been reportedly used for source authentication of gelatin. Nemati *et al.* (2004) investigated 14 porcine and five bovine gelatins using PCA of amino acid profile obtained through (RP) HPLC-fluorescence analysis of acid hydrolysis of gelatin. About 20 peaks were reportedly detected by HPLC analysis, of which one was very peculiar for bovine gelatin. PCA of peak height, area, area percentage and width was successfully used to differentiate gelatin based source. It was concluded that PCA of matrix height, width, and total matrix were capable of differentiation of bovine and porcine gelatins. Table 3 showed previous studies on Chromatographic-Chemometric analysis for differentiation of gelatin source.

In order to differentiate between capsule shells containing bovine and porcine gelatin, Widyaninggar *et al.* (2012) developed a method that involves chemometric analysis of HPLC-fluorescence detection profile of amino acids. Amino acids of gelatin in capsule shells were obtained by HCL-hydrolysis. The quantity of amino acids was dependent on the source of gelatin and PCA score plot of PC1 and PC2, respectively described 64.4% and 15.7%, variations between porcine and bovine gelatins. Hence, PCA of HPLC amino acid profile could be suitable for specie-specific gelatin authentication. Raraswati *et al.* (2013) studied differentiation of bovine and porcine gelatins in soft candy based on amino acid profiles and chemometrics. Separation of HCL derived amino acids was achieved by (RP) HPLC-fluorescent detector. Percentage of the peak height of the amino acids was analysed by PCA. PC1 and PC2 could be used to distinguish between porcine and bovine gelatins in soft candy. Azilawati *et al.* (2015) investigated chromatographic combined with a chemometric method to differentiate the bovine, porcine and fish gelatins. RP-HPLC-fluorescence method was used for separation of derivatives of HCL-hydrolysed gelatin samples. The PCA model demonstrated the relationships among amino acids in the correlation loadings plot to the group of gelatins in the scores plot. The method was stated to be very suitable for determination of gelatin from various sources.

In spite of all the above achievements, chromatographic-chemometric method has not been demonstrated yet to be capable for detection of contamination in gelatin containing mixture of different sources (Venien & Levieux 2005). Also, the possibility of transformation of amino acid through Maillard reaction during food processing might hinder the application of chromatographic-chemometric method for gelatin authentication.

### Chemisorption Method

Chemisorption is an adsorption process in which an adsorbate is held on the surface of an adsorbent by chemical bonds. Collagen, gelatin, and agarose gels stimulate the formation of hydroxyapatite (HAP) from amorphous calcium phosphate (ACP) (Termine *et al.* 1970). The difference in gelatin can result in their chemisorption behaviours. Hidaka and Liu (2003) investigated the possibility of authenticating gelatin sources (bovine bone or porcine skin) using chemisorption of ACP precipitate and promotion HAP formation. It was observed that both gelatins were ineffective on the rate of ACP formation and exhibited biphasic HAP transformation. Induction time from bovine bone gelatin differs from that of porcine gelatin and that the stimulatory effect of gelatinase hydrolysis of bovine bone gelatin on the rate of HAP transformation was eradicated. However, this method required further study on effect of different reaction conditions like temperature, pH and concentration. Also, this method will not be suitable to detect bovine and porcine gelatin in their mixture (Venien & Levieux 2005).

## CONCLUSION

We have reviewed various works on development of gelatin authentication methods. Most of the methods were stated to be very useful in food and pharmaceutical products. However, some accuracy and precision challenges have been identified. Gelatin similarities and possibility of process transformation of gelatins have been among the major challenges. Also, mass spectrometric method is very costly, long running time and required skilled personnel (Azilawati *et al.* 2015). Gelatin transformation during processing alone or in the presence of other food macro molecules can hinder the differentiation capability of present gelatin-source authentication methods. Future research works should focused on the aforementioned challenges.

## Figures and Tables

**Table 1:** Gelatin authentication methods and the tested samples.

Methods	Samples	References
Spectroscopic	Gelatin and gelatin hydrolyzates from bovine and porcine	Hashim *et al.* (2010); Hermanto & Fatimah (2013)
Immunochemical	Bovine and porcine skin and bone gelatin that has undergone acid or alkaline	Venien & Levieux (2005)
	Porcine gelatin in edible bird’s nests	Tukiran *et al.* (2015, 2016a)
	Gelatin sources in confectionery products	Tukiran *et al.* (2016b)
Nucleic acid based	Gelatin in the capsule	Cai *et al.* (2012); Malik *et al.* (2016); Mutalib *et al.* (2015); Sudjadi *et al.* (2015)
	Gelatin blends	Demirhan *et al.* (2012); Shabani *et al.* 2015)
	Marshmallow/cake, gum-drops, desserts, jelly and Turkish delight	
Mass Spectrometric	Bovine and porcine gelatin hydrolyzates	G.-F. Zhang *et al.* (2008); G. Zhang *et al.* (2009)
Electrophoretic analysis	Porcine type a and bovine type B gelatins	Azira *et al.* (2014); Hermanto & Fatimah (2013)
Chemosorption	Bovine bone and porcine skin gelatin	Hidaka & Liu (2003)
Chromatographic-chemometric	Bovine	Widyaninggar *et al.* (2012)
	Porcine and fish gelatins	Azilawati *et al.* (2015)
	Gelatin in capsule	Nemati *et al.* (2004)
	Gelatin in soft candy	Raraswati *et al.* (2013)

**Table 2:** Characteristics of bovine and porcine primers previously used in halal authentication of gelatin source.

Species	Primer sequence	Sensitivity	Product size	Genes	References
Porcine	5′-ATT TCC ATC CCA CAG CCC-3′	1%		MPRE42	Cai *et al.* (2012)
	5′-AAC AGA TGC TGA CTC ACA GAC-3′				
Bovine	5′-CTA AGA TCA TGG CAT CAG GTC C-3′				
	5′-CCC CAA AAT AAA GTC AGC CAC-3′				
Bovine	5′-GCCTAAATCTCCCCTCAATGGTA-3′	NA	271 bp	Cyt b	Shabani *et al.* (2015)
	5′-ATGAAAGAGGCAAATAGATTTTCG-3′				
Porcine	5′-GCCATATACTCTCCTTGGTGACA-3′		212 bp	Cyt b	
	5′-GTAGGCTTGGGAATAGTACGA-3′				
Porcine	5′-GAC CTC CCA GCT CCA TCA AAC ATC TCA TCT TGA TGA AA-3′	0.25 ng	398	Cyt b	Mutalib *et al.* (2015)
	5′-GCT GAT AGT AGA TTT GTG ATG ACC GTA-3′				
	5′-GCC TAA ATC TCC CCT CAA TGG TA-3′	0.1 ng	212	Cyt oxidase II	
	5′-ATG AAA GAG GCA AAT AGA TTT TCG-3′				
	5′-CTA CCT ATT GTC ACC TTA GTT-3′	0.0001 ng	83	ATP6	
	5′-GAG ATT GTG CGG TTA TTA ATG-3′				
Porcine	5′-CGT ATG CAA AAA ACC ACG CCA-3′	5 pg		D-Loop 108	Sudjadi *et al.* (2015)

*Notes*: MPRE42 = Meat Porcine Repetitive Element; Cyt = Cytochrome; NA = Not Available; ng = nanogram; pg = pico gram
